# Influence of Intraoperative Active and Passive Breaks in Simulated Minimally Invasive Procedures on Surgeons’ Perceived Discomfort, Performance, and Workload

**DOI:** 10.3390/life14040426

**Published:** 2024-03-22

**Authors:** Rosina Bonsch, Robert Seibt, Bernhard Krämer, Monika A. Rieger, Benjamin Steinhilber, Tessy Luger

**Affiliations:** 1 Institute of Occupational and Social Medicine and Health Services Research, Eberhard Karls University and University Hospital Tübingen, Wilhelmstraße 27, 72074 Tübingen, Germany; 2 Clinic for Hand, Plastic, Reconstructive and Burn Surgery, BG Clinic Tübingen, Schnarrenbergstraße 95, 72076 Tübingen, Germany; 3 Department of Gynecology and Obstetrics, University Hospital Tübingen, Calwerstraße 7, 72076 Tübingen, Germany

**Keywords:** laparoscopy, physical complaints, gynecology, surgeon well-being, surgical precision

## Abstract

Laparoscopic surgeons are at high risk of experiencing musculoskeletal discomfort, which is considered the result of long-lasting static and awkward body postures. We primarily aimed to evaluate whether passive and active work breaks can reduce ratings of perceived discomfort among laparoscopic surgeons compared with no work breaks. We secondarily aimed to examine potential differences in performance and workload across work break conditions and requested the surgeons evaluate working with passive or active work breaks. Following a balanced, randomized cross-over design, laparoscopic surgeons performed three 90 min laparoscopic simulations without and with 2.5 min passive or active work breaks after 30 min work blocks on separate days. The simulation included the following tasks: a hot wire, peg transfer, pick-and-place, pick-and-tighten, pick-and-thread, and pull-and-stick tasks. Ratings of perceived discomfort (CR10 Borg Scale), performance per subtask, and perceived workload (NASA-TLX) were recorded, and the break interventions were evaluated (self-developed questionnaire). Statistical analyses were performed on the rating of perceived discomfort and a selection of the performance outcomes. Twenty-one participants (9F) were included, with a mean age of 36.6 years (SD 9.7) and an average experience in laparoscopies of 8.5 years (SD 5.6). Ratings of perceived musculoskeletal discomfort slightly increased over time from a mean level of 0.1 to 0.9 but did not statistically significantly differ between conditions (*p* = 0.439). Performance outcomes of the hot wire and peg transfer tasks did not statistically significantly differ between conditions. The overall evaluation by the participants was slightly in favor regarding the duration and content of active breaks and showed a 65% likelihood of implementing them on their own initiative in ≥90 min-lasting laparoscopic surgeries, compared with passive breaks. Both passive and active breaks did not statistically significantly influence ratings of perceived discomfort or perceived workload in a 90 min simulation of laparoscopic surgery, with an overall low mean level of perceived discomfort of 0.9 (SD 1.4). As work breaks do not lead to performance losses, rest breaks should be tested in real-life situations across a complete working shift, where perceived discomfort may differ from this laboratory situation. However, in this respect, it is crucial to investigate the acceptance and practicality of intraoperative work breaks in feasibility studies in advance of assessing their effectiveness in follow-up longitudinal trials.

## 1. Introduction

Surgeons have to cope with multiple demands, including continuously developing their specialist knowledge, technical skills, and ability to cope with stress. Patient safety is of major concern before, during, and after surgical procedures because any surgical error may have catastrophic consequences [1,2]. Furthermore, surgery is a physically demanding profession where working in strenuous positions and under static loads is common [3,4]. The often-resulting high mental and physical workloads were found to be associated not only with negative impacts on the surgeon but also on patients, who are at increased risk for complications and prolonged hospital stays [5]. Kc and Terwiesch [6] investigated patient safety and concluded that increased physician workloads led to increased in-hospital and post-discharge mortality rates due to early discharges. Therefore, it is worthwhile to improve working conditions as much as possible [7].

In recent studies, 19% of surgeons reported experiencing musculoskeletal symptoms or discomfort due to their work [8], with laparoscopic surgeons at the highest risk, ranging from 55% to 88% [9,10,11,12]. One approach on the part of occupational physiology is work-related organizational interventions for reducing work-related musculoskeletal disorders. In this respect, short rest periods during mental and physical tasks as well as a reduction in working hours are interventions known to result in improvements in terms of quality and quantity of work [13]. In other occupational fields, lack of rest periods, captured by the factor “time pressure,” showed a statistically significant positive association with long-term sick leave (>10 days) [14]. Also in sports, breaks, also known here as time-outs, are already established and influence the performance afterwards [15].

Short breaks during surgery are easy to implement, from which not only the surgeon but the entire operating team benefits. In several studies, intraoperative breaks of 1.5–5 min implemented every 20–40 min resulted in decreased discomfort in the back and neck [16,17] and shoulders [17,18,19] and decreased stress-related cortisol in the saliva [18] of the surgeons. Note that microbreaks as short as 10 s implemented every 10 min did not show effects on the surgeon’s performance or well-being [20]. With regard to patient well-being, the 1.5–5 min lasting breaks had no significant effect on the duration of surgical procedures performed [18,19].

Regarding surgeon performance, the effects of breaks on work precision have already been investigated in two clinical trials. In a study with surgical procedures lasting ≥2 h with 20 s breaks every 20 min, the error rate was lower in the intervention group [17]. In a study of surgeries lasting <1 h with 30 s breaks every 15 min, no difference was observed in the precision task performed [21]. In both studies, precision was compared before and after surgery using non-laparoscopic precision tasks. During laparoscopic surgery, the assessment of performance in the clinical setting is difficult and has not been reported so far.

The studies conducted to date compared either active work breaks with non-work-related cognitive or physical content or passive work breaks without any tasks with a control condition without breaks. The primary aim of this study was to determine whether passive and active work breaks would reduce ratings of perceived discomfort among gynecologists performing simulated laparoscopic procedures compared with no work breaks. Our secondary aim was to examine possible differences in work performance and perceived workload among the conditions without passive and active work breaks. A standardized procedure under comparable working conditions was required, and a laboratory approach was chosen. Further secondary outcome parameters (i.e., muscular activity, muscular fatigue, upper body posture, heart rate, and heart rate variability) are described in Luger et al. [22].

## 2. Materials and Methods

### 2.1. Study Design

We conducted a cross-over randomized controlled trial in laboratory settings from March 2019 to October 2020, registered under NCT03715816 [23], following the ethical standards of the Declaration of Helsinki [1]. Each participant underwent the control condition without breaks and the two intervention conditions with passive and active breaks. The order was determined by a random allocation by drawing a lot to one of the six possible sequences; blinding of the study participants was not possible due to the character of the experiment. In addition, the statistical analysis and the evaluation of the results were not blinded either.

### 2.2. Study Sample

With perceived discomfort as primary outcome, sample size was calculated using the equation of Kadam and Bhalerao [24]. Underlying results from Dorion and Darveau [17] (effect size 1.6857; pooled SD 1.9337), a study power of 80% and a level of significance of 5% resulted in a required sample size of 21 without consideration of potential drop-outs.

For participation, subjects had to fulfill the following eligibility criteria: sufficient knowledge of German language, being 18 years of age or older, experience working laparoscopically, and successfully completing the Peg transfer task (i.e., <3 min) [25]. Surgically experienced, currently employed subjects were recruited via internal mailing lists and direct advertisement at the Department of Gynecology and Obstetrics at the University Hospital Tübingen.

### 2.3. Intervention

According to the existing findings of previous studies and in coordination with the deputy director and senior surgeon in the department where we recruited our subjects (details on the theoretical approach, see [26,27]), the duration of the simulated operation (90 min) as well as the length (2.5 min) and frequency (every 30 min) of the break-intervention were determined. Participants performed the 90 min simulated laparoscopy, consisting of three repetitive 30 min work blocks in a controlled laboratory environment, three times: (1) a control condition, during which the participant worked continuously, i.e., similar to usual working conditions; (2 & 3) two intervention conditions, within which 2.5 min passive or active breaks were introduced after each 30 min work block. During both types of breaks, laparoscopic instruments were laid down. While during passive breaks participants rested in standing position [28], during active breaks exercises were performed. The exercise protocol consisted of eight tasks aiming at normalization of tissue tension, posture correction, and relaxation [29] in the mainly affected body regions of neck, shoulders, and lower back [30,31] and were guided by a standardized audio file (transcribed protocol, see Supplementary Materials Scheme S1). Range of motion and number of repetitions of each exercise were not controlled. The exercises performed during active breaks were designed in collaboration with a physical therapist and sports scientist.

### 2.4. Experimental Procedure

At first, we met each participant in the laboratory for a familiarization trial. After informing participants about the procedure of the experiment and the aims of the study and answering all open questions, all necessary participation documents were filled out, and the participant gave written, informed consent. After meeting all necessary criteria, all participants could proceed to practicing the laparoscopic tasks and getting used to the laparoscopic instruments. The active break exercises were explained and practiced following the audio instructions. Furthermore, a briefing on the question of rating perceived physical discomfort took place. Finally, data concerning demographics and musculoskeletal status were gathered using the German version of the standardized Nordic Musculoskeletal Questionnaire [32]. All experimental conditions, as well as the familiarization trial, took place on separate days.

The simulated laparoscopic tasks were performed in a Pelvic Trainer (Szabo, ID Trust Medical, Leuven, Belgium) with fastened light source and optics (Karl Storz 26003AA HOPKINS II Optik 0°, Karl Storz SE & Co. KG, Tuttlingen, Germany) and visualization on a suitable monitor (WideView Monitor 26″ HD, SC-WU26-A1515, Karl Storz SE & Co. KG, Tuttlingen, Germany). The slightly right-lateral monitor position was individually adjusted, reaching an ~10° ergonomically sound downward view to avoid neck extension [33] at a distance of ~80 cm from the standing workplace of the subject. As instruments for bimanual working, a standardized combination of two instruments was used: in the right hand, a 33 cm long multifunctional forceps with 1:2 teeth (RS225-595, RUDOLF Medical GmbH & Co. KG, Fridingen, Germany); in the left hand, a 36 cm long grasping forceps without teeth (33321 KW, Karl Storz SE & Co. KG, Tuttlingen, Germany). For the task pick-and-thread, the instrument on the right hand was replaced by a 33 cm long needle holder (26173 KAR KARL STORZ SE & Co. KG, Tuttlingen, Germany). The hot wire task was executed with the right hand only using a Maryland bipolar forceps (20195-225, ERBE Elektromedizin GmbH, Tübingen, Germany). The working conditions were standardized, such as light intensity (5–9 lx) and room temperature (24–26 °C); further information is provided in the study protocol [27].

**Figure 1:**
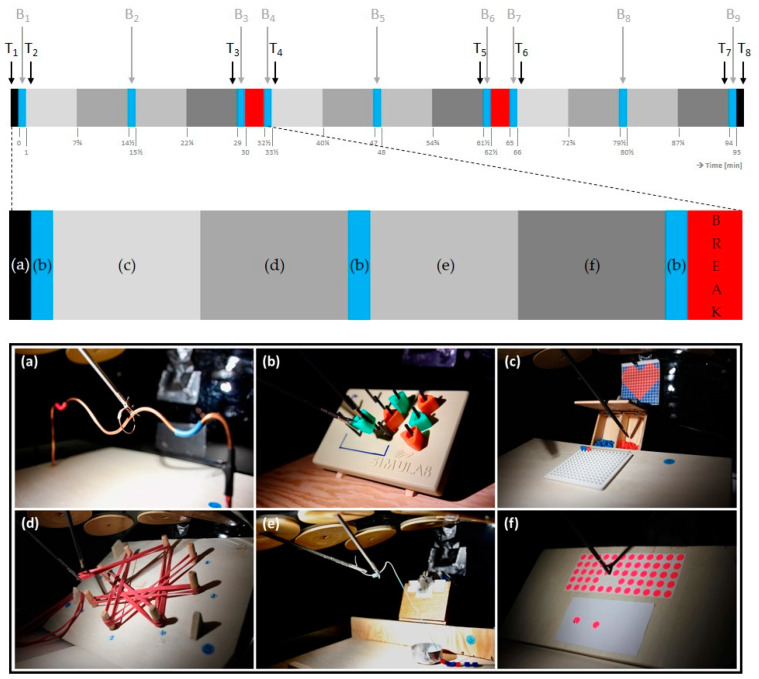
The time course of an experimental condition; in the control condition, there will be no breaks (red). Each block and letter is related to a task, including the (**a**) hot wire, (**b**) peg transfer, (**c**) pick-and-place, (**d**) pick-and-tighten, (**e**) pick-and-thread, and (**f**) pull-and-stick task. The arrows indicate the time points of recordings of ratings of perceived musculoskeletal discomfort (T_1–8_) and performance during the peg transfer task (B_1–9_).

All three 30 min work blocks of the 90 min simulated laparoscopy were equally structured. The task selection and execution sequence were determined by the profile of ability requirements assessed by the authors and a specialist in general, visceral, and trauma surgery (task ability requirements, see Supplementary Materials Table S1). In four 6.75 min lasting working sections, the tasks pick-and-place (beads), pick-and-tighten (gummies), pick-and-thread (beads), and pull-and-stick (stick-on points) were performed in this given order. Simulating the food-hand coordination necessary for laparoscopy bipolar coagulation, a foot pedal was used during the pick-and-place and pick-and-thread tasks to open a box that contained the needed beads. At the beginning and end of the 30 min work blocks, as well as in the middle between the second and third work blocks, the participants performed the 1 min peg transfer task. Before and after the simulated laparoscopy, a hot wire task was performed. Tasks and sequence of the simulation operation are schematically shown in Figure 1. Further information about all tasks is available in the study protocol [27].

### 2.5. Data Collection and Data Analysis

Data collection and analysis of additional secondary outcome parameters, including muscular activity, muscular fatigue, upper body posture, heart rate, and heart rate variability [27], are reported in another results paper [22]. The primary outcome, i.e., rating of perceived discomfort, was chosen because it is a recognized predictor of musculoskeletal disorders [34].

#### 2.5.1. Rating of Perceived Discomfort

Perceived discomfort was assessed verbally at eight time points, i.e., at the beginning and end of the three 30 min work blocks and before and after the simulated laparoscopy (Figure 1). If complaints were present (“Do you have any discomfort”), the participant rated general perceived discomfort (“Please indicate the intensity of the current discomfort”) on an eleven-point numerical rating scale [35] from 0 (no discomfort at all) to 10 (maximum imaginable discomfort), after which the location according to the body map of Corlett [36] was assessed (“Please indicate in which parts of your body you feel discomfort”).

#### 2.5.2. Performance

All six tasks in the simulation operation allow for quantification of the performance achieved. The four tasks in the 6.75 min work sections and the peg transfer task each yield a number of transferred objects. For the peg transfer, the time to complete one pass (6 prisms) was additionally determined. With regard to the hot wire task, the time required to complete it as well as the relative proportion of the completion time [%] in which the hot wire was not touched were determined. Performance outcomes of the hot wire and peg transfer tasks were statistically analyzed; other performance outcomes are presented descriptively.

#### 2.5.3. Perceived Workload

After each experimental condition, participants completed the National Aeronautics and Space Administration Task Load Index (NASA-TLX) to record perceived workload for each of the performed tasks (cf. Figure 1). It comprises six dimensions of mental demand, physical demand, temporal demand, own performance, effort, and frustration, which were asked on a scale of 0 to 100 for each of the six tasks performed [37]. The overall workload was determined as the unweighted average [38] of all six dimensions and all six tasks, ranging between 0 (low subjective workload) and 100 (high subjective workload).

#### 2.5.4. Subjective Evaluation of the Implemented Breaks

Participants were asked to rate each break intervention in terms of content, frequency, duration, and its impact on surgeons’ concentration and performance on a five-point Likert scale. The body region(s) within which a recovery effect was perceived was indicated. Finally, participants were asked to rate the likelihood of incorporating breaks into their daily surgical routine on a 100-mm visual analogue scale ranging from absolutely unlikely (left; 0) to absolutely likely (right; 100). The questionnaire was filled out after both the active and passive intervention conditions (see Supplementary Materials Scheme S2).

### 2.6. Statistical Analysis

A randomized cross-over design with 21 subjects was chosen to determine whether active or passive pause during laparoscopic procedures was more likely to provide benefits in terms of surgeon discomfort. We inspected whether the outcome parameters were normally distributed [39] and had to conclude that none of them were. Therefore, data are presented as medians with interquartile ranges in boxplots. All statistical analyses were performed using SPSS software (IBM^®^ Statistics^®^ 28.0, Armonk, NY, USA) and JMP software (JMP^®^ 16.2.0, SAS Inc., Cary, NC, USA) at a significance level of 5% (*p* = 0.05).

We performed generalized estimating equations (GEE) to test the within-subject effects of work break condition (three levels: no, passive, active breaks) and time (two time points of interest: shortly after the start B_1_ and shortly before the end of the simulated operation B_9_) on both the primary outcome and secondary outcomes. GEE was used because it permits analyzing non-normal distributions as well [40]. Characteristics of the GEE response variable were adapted according to the distribution characteristics of each outcome measure.

For the primary outcome of perceived discomfort, we used GEE with an exchangeable correlation matrix and gamma scale response to test the within-subject effects of work break condition and time. For the secondary outcomes on performance, we used GEE with an exchangeable correlation matrix and Normal scale response (i.e., number of transferred pegs in 60 s), Gamma scale response (i.e., peg transfer task completion time for one pass of six pegs, hot wire task completion time), or inverse Gaussian scale response (i.e., proportion of time the wire was not touched) to test the within-subject effects of work break condition and time. In cases of significant main or interaction effects, we performed Šidák for post hoc pairwise comparisons. We calculated effect sizes for main and interaction effects using Cohen’s index *w* and for pairwise comparisons using Cohen’s *d* (average SD of both comparators as standardizer) [41] and interpreted them as small (*w* ≥ 0.1; *d* ≥ 0.2), medium (*w* ≥ 0.3; *d* ≥ 0.5), or large (*w* ≥ 0.5; *d* ≥ 0.8) [42]. The secondary outcomes of workload (NASA-TLX) and the subjective evaluation questionnaire were analyzed descriptively.

#### Exploratory Subgroup Analyses

Following an exploratory approach, we investigated the effect of work breaks on the primary outcome across various subgroups, including sex, age, job tenure, and previous work-related musculoskeletal symptoms. We split the dataset in two for each subgroup parameter: sex (men vs. women), age (younger ≤ 35 years vs. older > 35 years), job tenure (novices ≤ 6 years vs. experts > 6 years), and history of work-related musculoskeletal symptoms in the past twelve months (yes vs. no as reported on the Nordic Musculoskeletal Questionnaire). We then performed the GEE for perceived discomfort again, as described previously.

## 3. Results

Descriptive statistics are summarized in Table 1, and the results of the statistical analyses are summarized in Table 2. The results of the exploratory subgroup analyses are available in Supplementary Materials Tables S2 and S3, and Figure S1.

### 3.1. Study Sample

Twenty-five subjects were recruited, of whom four dropped out (lack of time, change of employer and moving away, spine injury, traumatic injury to the clavicula). The final sample of twenty-one subjects (9F, 12M) included two participants with <2 years, nine participants with 4–7 years, and ten participants with ≥9 years of experience in laparoscopic surgery. Seventeen subjects had full-time and four part-time jobs with an average of 44 work hours per week (SD 20). The average age of the 21 right-handed subjects was 36.6 years (SD 9.7; range 22–63). Fifteen of the 21 subjects reported to exercise regularly with an average of 3.9 h/week (SD ±2.5; range 1.5–10), in particular endurance-related sports.

Musculoskeletal discomfort in the past 12 months and past 7 days [32] was reported by 16 participants (76.2%). Particularly affected body regions were the lower back (47.6% in the last 12 months; 14.3% in the last 7 days), neck (33.3% in the last 12 months; 14.3% in the last 7 days), shoulder (28.5% in the last 12 months; 9.5% in the last 7 days), and upper back (23.8% in the last 12 months; 14.3% in the last 7 days).

The final dataset included a few missing values from three different subjects. The completion time of the peg transfer task was missing at B_1_ of the passive break condition due to failed manual recording (subject 1), the success rate of the hot wire task was missing before the active break condition due to a failure in the audio recording (subject 2), and NASA-TLX temporal demand, effort, and performance were missing for the peg transfer task in the active break condition due to no answers/crosses provided (subject 3).

### 3.2. Rating of Perceived Discomfort

**Figure 2:**
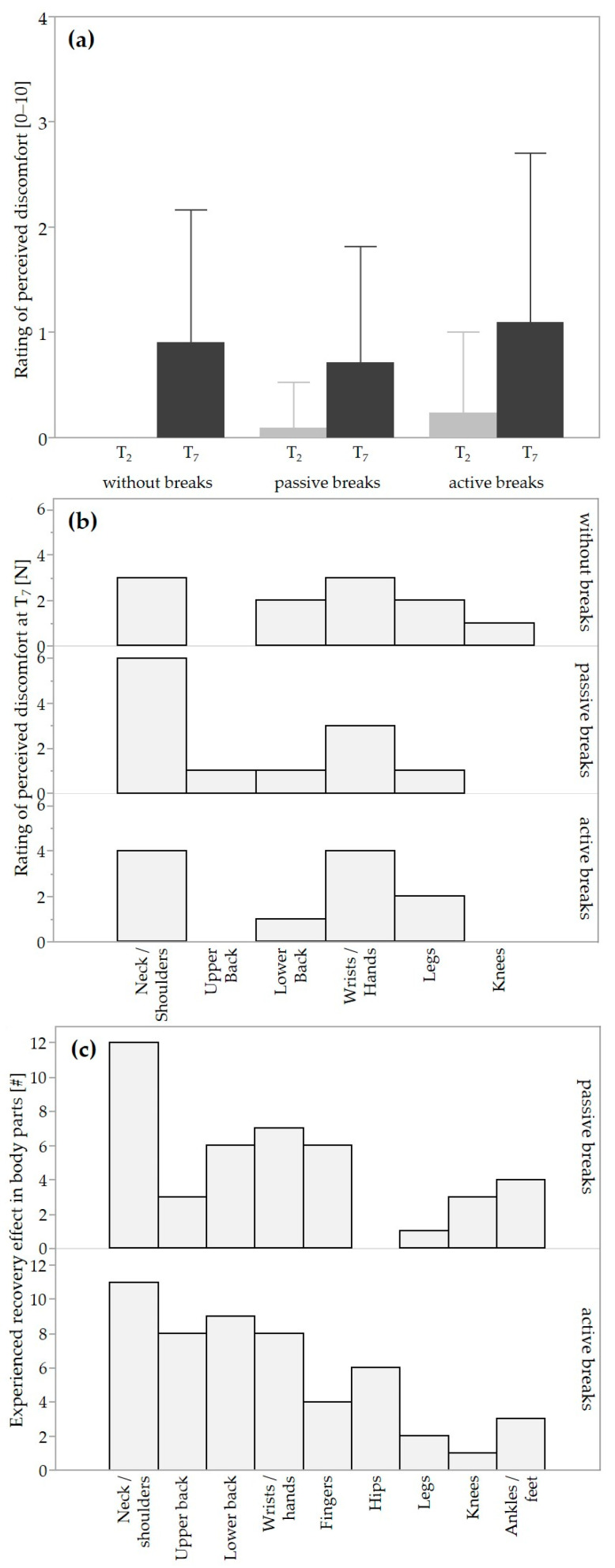
Rating of perceived discomfort and experienced recovery effect: (**a**) the intensity of the rating of perceived musculoskeletal discomfort in the beginning (T_2_, light gray) and at the end (T_7_, dark gray) of the experimental condition as mean ± SD; (**b**) histogram showing the number of subjects reporting musculoskeletal discomfort in various body regions at T_7_; (**c**) histogram showing the number of subjects (#) experiencing a recovery effect due to the work break intervention.

During the simulated laparoscopies, 12 out of 21 participants (57.1%) experienced discomfort in at least one experimental condition at a total of 119 (23.6%) of the 504 interrogation time points. When complaints were present, they ranged from 1 to 5 on the NRS (minimum = 0, maximum = 10), with means ranging from 0.7 (passive breaks) to 0.9 (without breaks) to 1.1 (active breaks). The performed GEE revealed no statistically significant main effect of condition (*p* = 0.439; *w* = 0.114) or interaction effect (*p* = 0.101; *w* = 0.191), but a statistically significant main effect of time (*p* < 0.01) with medium effect size (*w* = 0.311). The rating of perceived discomfort increased over time in all three conditions by ~0.8 points, on average (Figure 2a). At T_7_, the most frequently affected body regions were the neck and shoulders and the wrists and hands (Figure 2b).

The subgroup analysis on sex revealed a comparable effect of time, i.e., the rating of perceived discomfort significantly increased for both women and men. However, women showed a statistically significant interaction effect (*p* = 0.027), where the perceived discomfort rating statistically significantly increased over time on average from 0.3 to 1.6 for the active work breaks only. The subgroup analysis on age revealed that the effect of time was only statistically significant among the younger (*p* < 0.01; 0.1 vs. 1.2) and not among the older laparoscopists (*p* = 0.071; 0.1 vs. 0.6). The subgroup analysis on job tenure revealed that the effect of time was only statistically significant among the novices (*p* = 0.002; 0.1 vs. 1.4) and not among the more experienced surgeons (*p* = 0.060; 0.1 vs. 0.5). The subgroup analysis on previous work-related musculoskeletal symptoms could not be performed because the group without previous work-related musculoskeletal symptoms was too small (N = 5).

### 3.3. Performance

**Figure 3:**
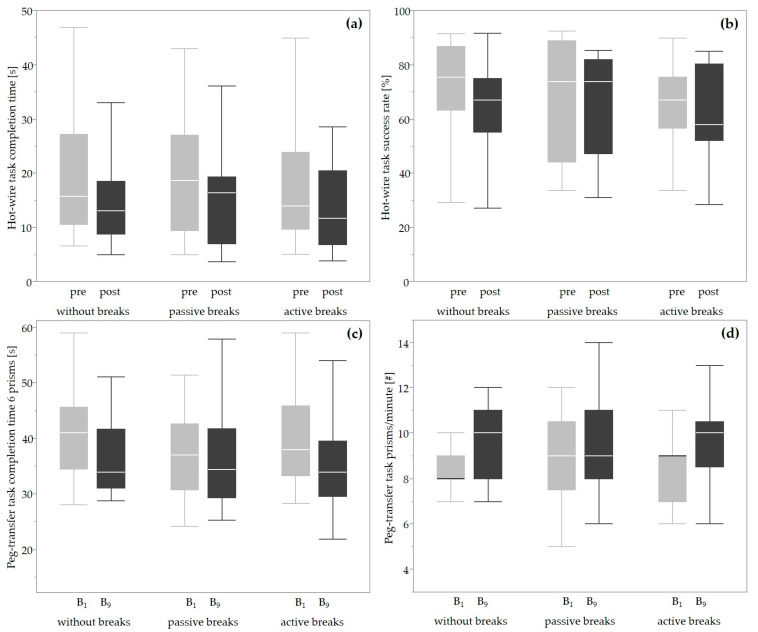
Boxplots displaying the minimum, first quantile, median, third quantile, and maximum of the outcomes. Hot wire task performance pre- (light gray) and post- (dark gray) conditions: (**a**) completion time; (**b**) success rate. Peg transfer task performance at the beginning (B_1_, light gray) and at the end (B_9_, dark gray) of the condition: (**c**) completion time of six prisms; (**d**) number of transferred prisms (#).

None of the four outcomes related to the hot wire and peg transfer tasks revealed statistically significant main effects of condition (*p* > 0.110) or interaction (*p* > 0.150). Statistically significant main effects of time (*p* < 0.01) were present for hot wire task completion time (*w* = 0.387), completion time of six prisms (*w* = 0.411), and number of prisms in sixty seconds (*w* = 0.498). The completion time of the hot wire task (Figure 3a) and transferring six prisms (Figure 3c) decreased by 2.0 s (IQR 5.8) and 3.7 s (IQR 8.0), respectively, and the number of transferred prisms within 60 s (Figure 3d) increased by 1.0 (IQR 2.0) over time.

The median performance of the four experimental tasks (Supplementary Materials Figure S2) tended to slightly increase over time within all three conditions. However, comparing the conditions, their medians are rather similar and not likely to be different.

### 3.4. Perceived Workload

The median workload was 47.6 (IQR 11.7) and did not differ much across conditions. The overall workload was perceived as lowest for the peg transfer task (median 35.0, IQR 18.3) and highest for the hot wire task (median 59.2, IQR 26.7). Each of the six dimensions contributed equally to the overall workload (i.e., unweighted average); effort was characterized by the highest median value of 51.7 (IQR 16.7) and frustration by the lowest median value of 40.0 (IQR 16.7). All six workload dimensions are visualized in Supplementary Materials Figure S3.

### 3.5. Subjective Evaluation of the Implemented Breaks

**Figure 4:**
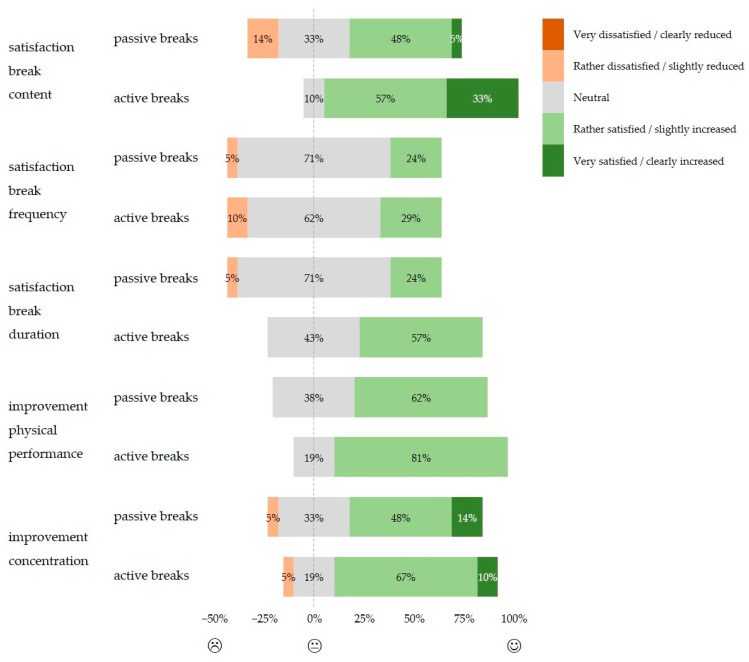
Results of the relative portions [%] of the five Likert scale questions as part of the evaluation questionnaire for the passive and active work breaks. Answer categories for each of the five Likert scale questions can be found in Supplementary Materials Scheme S2.

The body parts in which most participants experienced recovery during both the passive and active work breaks were the neck and shoulders (N = 11–12; Figure 2c), followed by the hands (N = 7), lower back (N = 6), and fingers (N = 6) during the passive, and followed by the lower back (N = 9), upper back (N = 8), hands (N = 8), and hips (N = 6) during the active work breaks. The three aspects of content, frequency, and duration of both types of work breaks were generally positively rated, with a more positive balance for active work breaks regarding content and duration (Figure 4). The evaluation of one’s own physical performance and concentration ability were generally positively rated for both passive and active work breaks (Figure 4). The median likelihood of taking short work breaks at their own initiative during a routine surgery lasting at least 1.5 h equaled 6.7 (IQR 4.9) for passive and 6.5 (IQR 4.6) for active work breaks, translated as rather likely. Note that the subjective evaluations covered the whole range from 0, very unlikely, to 10, very likely.

## 4. Discussion

Our primary aim was to determine whether passive and active work breaks would reduce ratings of perceived discomfort among gynecologists performing simulated laparoscopic procedures compared with no work breaks. The hypothesis was not confirmed with perceived discomfort ratings at the end of the simulation ranging (mean) from 0.7 (passive) to 0.9 (without) and 1.1 (active), which were not statistically significantly different from each other (*p* = 0.439). Our study population showed a positive attitude towards active breaks plus a 65% likelihood of implementing self-initiated active work breaks during laparoscopies lasting ≥90 min, which is in line with the recommendations of recent overview articles [43,44]. Our secondary aim was to examine potential differences in work performance and perceived workload among the conditions without passive and active work breaks. The results do not indicate any relevant differences between conditions for work performance, since box plots are mainly overlapping each other (Figure 3 and Supplementary Materials Figure S2), nor for perceived overall workload (medians ranging from 47.1 for without to 47.6 for passive/active; Supplementary Materials Figure S3).

### 4.1. Rating of Perceived Discomfort

We observed that 57% of the participants reported musculoskeletal discomfort, which confirms numbers reported by previous studies that ranged from 55 to 88% [9,10,11,12]. As was to be expected, the rating of perceived discomfort increased by ~0.8 points during the simulated laparoscopy (Figure 2a). For sample size calculation, the effect size and pooled standard deviation for the measure of perceived discomfort were assumed to be 1.6857 and 1.9337, respectively, based on a study where the authors investigated intraoperative 20 s active work breaks every 20 min during surgeries lasting at least 120 min compared with no work breaks [17]. Yet, comparable effects could not be identified in the present study, i.e., no differences between the intervention and control conditions could be observed, which might be due to the simulated setting that resulted in relatively low discomfort values ranging from 0.7 to 1.1. As a comparison, studies conducted in the surgical field reported generally higher levels of discomfort under standard conditions without intraoperative breaks, i.e., 3/10 in neck and 5/10 in lower back during open surgeries [17], and 2.5/10 in neck and 3/10 in lower back in laparoscopic surgeries [18].

The body regions that were most affected by experiencing discomfort in the current study (i.e., neck and shoulders; Figure 2b) corresponded to the body regions as reported in studies [30,31,45,46]. With the implementation of both types of work breaks, study participants felt the greatest recovery effect in the neck and shoulders and, to a lesser extent, also in the back, wrists, and hands. These body regions were also reported by previous studies to be positively affected by the implementation of work breaks [16,17,18,19]. This advocates that the implementation of work breaks over the long term may have a positive impact on the incidence of discomfort experienced in the aforementioned body regions.

Taking age and job tenure into account may contribute to a better assessment of the effectiveness of intraoperative breaks on ratings of perceived discomfort. The results of the exploratory subgroup analyses showed that the younger and novice laparoscopists (1.2 and 1.4, respectively) experienced higher average levels of discomfort by the end of the simulated laparoscopies compared with the older and expert laparoscopists (0.6 and 0.5, respectively). Previous studies are ambiguous regarding associations between age and job tenure and the prevalence of WRMSD. For age, there are results pointing to an increased reported prevalence of WRMSD in the aging workforce [47,48]. However, studies that focused on health care providers showed that age had no statistically significant association with the prevalence of WRMSD [49,50,51]. For job tenure, Yizengaw, Mustofa, Ashagrie, and Zeleke [49] showed that health care providers with less than five years of experience had a somewhat lower prevalence of WRMSD (62%) than those with five or more years of experience (71%). Contrariwise, Boyer, Galizzi, Cifuentes, d’Errico, Gore, Punnett, and Slatin [50] showed that health care providers with less than two years of experience had a much higher risk of WRMSD (41%; RR = 4.8) than those with two or more years of experience (13%). It should be noted that age and work experience overlap, as less experienced individuals also tend to be of younger age than more experienced individuals. The subgroup analysis of sex did not reveal any relevant differences between women and men regarding perceived discomfort ratings, which is in line with previous studies reporting rather similar prevalence rates of WRMSD for female and male health care workers [49,50,52]. However, other studies reported a higher prevalence of WRMSD among females compared with males [47,51,53]. Note that the prevalence rates of WRMSD for women and men may, to a large extent, be related to differential exposure to the type of work and related workload [53].

### 4.2. Performance

We noticed that the hot wire task was performed faster but more inaccurately with time (cf. Figure 3), which may point to a supposed loss of concentration with time. However, the performance of any of the six simulated tasks did neither improve nor worsen with the implementation of work breaks. In contrast, the perceived performance as one dimension judged by answering the NASA-TLX questionnaire tended to be slightly reduced for both passive (50.0) and active (48.3) work breaks when compared with the condition without (40.0) breaks. Contrary to this, 81% (N = 17) and 62% (N = 13) of the participants perceived physical performance (as part of the subjective assessment questionnaire) to be improved with the implementation of passive and active work breaks, respectively. These numbers are in line with previous findings, where 57% [19] and 92% [54] of the participants reported improved physical performance after the implementation of active work breaks.

### 4.3. Perceived Workload

The overall workload (NASA-TLX) among the surgeons in this study (47.6) corresponds very well (47.5) to that from the study of Krämer et al. [55], and is close to those of the studies of Thurston et al. [56] and Haney et al. [57] (i.e., 40.6 and 50.4, respectively). We may interpret the current study to have reached a similar moderate overall workload as the three previous studies [55,56,57]. The pattern of the six workload dimensions strongly agreed with a previous study in which 40 surgeons were exposed to a simulated laparoscopic that lasted about 26 min [57] and reported the lowest mean value for frustration (40.0) and the highest mean value for effort (51.7) as dimensions of perceived workload. However, two other studies showed a different pattern, with performance (25.8) as the dimension with the lowest and mental demand (50.3) as the dimension with the highest mean value [56] or temporal demand (20.0) as the dimension with the lowest and performance (90.0) as the dimension with the highest mean value [55]. These variations across studies may be due to the fact that simulations [57] have a different perceived workload exposure pattern than real laparoscopies [55,56]. The reason for this is that different work system factors may influence the perception of the different workload dimensions, such as team coordination, task content, availability of medical tools, and work station design [38,58].

### 4.4. Subjective Assessment of the Implemented Breaks

Our study participants evaluated the active work break condition slightly better considering its content and duration than passive work breaks (cf. Figure 4) and provided a 65% likelihood of implementing active work breaks on their own initiative in routine surgeries lasting ≥1.5 h. Even stronger positive evaluations were reported by Hallbeck, Lowndes, Bingener, Abdelrahman, Yu, Bartley, and Park [19], with 87% of the surgeons interested in implementing active work breaks in their operating room routine. The positive evaluations of the current study complement previous study findings, which reported that intraoperative breaks reduce upper extremity physical strain and stress [16,17,18,19,59]. These findings advocate for further investigation of active work break interventions for surgeons, e.g., a cluster randomized controlled trial including both subjective and objective outcome measures. Such follow-up studies can additionally provide insight into the acceptance and practical implementation of active work breaks with varying surgery durations.

### 4.5. Study Implications, Strengths, and Limitations

The standardized and controlled study procedure, i.e., 2.5 min breaks implemented after 30/60 min in 90 min simulated work blocks, contributed to a high internal validity. The design of the breaks regarding content, frequency, and duration was based both on previous study results as well as alignment with the deputy director and senior surgeon of the recruited department. Furthermore, the control condition (i.e., without breaks) reflected regular, realistic working conditions, and the range of motion and number of repetitions of the active break intervention were not controlled. These aspects led to a higher level of external validity, increasing the potential for future implementation of (active) work breaks. Moreover, if such intraoperative work breaks are implemented in real laparoscopic routines, other medical staff besides the main laparoscopic surgeon may benefit as well if the breaks are designed and implemented accordingly.

Some study limitations could also be recognized, meaning that, when interpreting the current results, one should bear in mind that this study (1) was performed under controlled laboratory conditions with strictly timed breaks being a challenge in real laparoscopies, (2) the chosen setting (laboratory and simulation tasks) was not suitable to induce relevant perceived discomfort in the majority of the subjects compared with discomfort levels reported in real surgical procedures [17,18], (3) included the main surgeon only instead of a more complete operating room team, (4) included a sample of 21 surgeons belonging to a single hospital department, (5) did not reach levels of discomfort that can be reached in real laparoscopic procedures where surgeons additionally deal with real patients and surgical interruption (e.g., phone call [60]) and (6) did not include potential covariates including sex, age and laparoscopic work experience but rather investigated them in exploratory post-hoc subgroup analyses.

## 5. Conclusions

A highly controlled laboratory simulation of laparoscopy revealed that both passive and active intraoperative work breaks did not significantly influence ratings of perceived discomfort. Overall, the active breaks were evaluated positively with respect to their content, frequency, and duration, leading to a considerable likelihood (65%) of implementing self-initiated active breaks during laparoscopic procedures lasting ≥90 min. Consequently, the impact of implementing active work breaks in routine laparoscopies on its acceptability, practicality, and effectiveness among surgeons and other medical staff remains to be investigated in detail in future feasibility studies and longitudinal (cohort) randomized controlled trials, also considering potential confounding effects of age, work experience, sex, and history of musculoskeletal complaints.

## Figures and Tables

**Table 1:** Descriptive results of rating of perceived discomfort (RPD), hot wire task performance, peg transfer task performance, performance of the four experimental tasks, and overall workload for the complete 90 min simulation and for each experimental task. Values are provided for each of the three experimental conditions and, if relevant, per follow-up assessments, i.e., right before (pre) or after (post) the condition, during the first/ninth peg transfer repetition (B_1_/B_9_), at minute 2/88 (T_2_/T_7_), over the first/second/third work block (WB_1_/WB_2_/WB_3_).

Task	Parameter	Without Breaks			Passive Breaks			Active Breaks		
		Pre/B_1_/T_2_/WB_1_	WB_2_	T_7_/B_9_/WB_3_/Post	Pre/B_1_/T_2_/WB_1_	WB_2_	T_7_/B_9_/WB_3_/Post	Pre/B_1_/T_2_/WB_1_	WB_2_	T_7_/B_9_/WB_3_/Post
RPD	Median (IQR)	0.00 (0.00)	-	0.00 (2.00)	0.00 (0.00)	-	0.00 (2.00)	0.00 (0.00)	-	0.00 (2.50)
	Mean (SD)	0.00 (0.00)	-	0.90 (1.26)	0.10 (0.44)	-	0.71 (1.10)	0.24 (0.77)	-	1.10 (1.61)
Hot wire	CT (s)	15.72 (16.67)	-	13.10 (9.78)	18.66 (17.71)	-	16.41 (12.75)	13.93 (14.21)	-	11.66 (13.48)
	SR (%)	75.48 (23.59)	-	67.08 (19.79)	73.91 (44.65)	-	73.89 (34.73)	67.08 (18.90)	-	57.98 (28.19)
Peg transfer	CT/6 prisms (s)	41.00 (11.12)	-	33.93 (10.53)	37.01 (11.74)	-	34.38 (12.44)	37.93 (12.61)	-	33.93 (9.98)
	Prisms/60 s (#)	8.0 (1.0)	-	10.0 (3.0)	9.0 (3.0)	-	9.0 (3.0)	9.0 (2.0)	-	10.0 (2.0)
Pick-and-place	Beads/6.75 min (#)	22.0 (7.0)	24.0 (6.5)	23.0 (7.5)	20.0 (7.5)	23.0 (6.5)	23.0 (8.5)	22.0 (10.5)	22.0 (5.5)	21.0 (7.0)
Pick-and-tighten	Gummies/6.75 min (#)	22.0 (4.0)	22.0 (3.5)	23.0 (4.0)	22.0 (6.0)	22.0 (6.5)	23.0 (7.0)	22.0 (6.5)	26.0 (7.5)	25.0 (6.5)
Pick-and-thread	Beads/6.75 min (#)	11.0 (8.0)	11.0 (7.5)	14.0 (6.0)	11.0 (6.5)	13.0 (6.0)	13.0 (8.5)	9.0 (7.5)	12.0 (9.5)	13.0 (5.0)
Pull-and-stick	Stick-on points/6.75 min (#)	23.0 (11.5)	26.0 (11.5)	26.0 (13.5)	24.0 (10.0)	27.0 (13.0)	30.0 (11.5)	28.0 (11.5)	30.0 (16.5)	32.0 (10.0)

IQR interquartile range; SD standard deviation; CT completion time; SR success rate; # number.

**Table 2:** Statistical results of the GEE and effect size index *w* for the main and interaction effects of rating of perceived discomfort (RPD), hot wire task performance, and peg transfer task performance.

Task	Parameter	Condition			Time			Condition × Time		
		χ^2^ (df)	*p*	*w*	χ^2^ (df)	*p*	*w*	χ^2^ (df)	*p*	*w*
-	RPD	1.646 (2)	0.439	0.114	12.198 (1)	0.000 *	0.311 ^†^	4.594 (2)	0.101	0.191
Hot wire	CT (s)	1.139 (2)	0.566	0.095	18.867 (1)	0.000 *	0.387 ^†^	2.196 (2)	0.334	0.132
	SR (%)	4.295 (2)	0.117	0.185	3.602 (1)	0.058	0.170	0.707 (2)	0.702	0.075
Peg transfer	CT/6 prisms (s)	3.521 (2)	0.172	0.168	21.078 (1)	0.000 *	0.411 ^†^	3.489 (2)	0.175	0.167
	Prisms/60 s (#)	1.312 (2)	0.519	0.102	31.203 (1)	0.000 *	0.498 ^†^	0.885 (2)	0.642	0.084

CT completion time; SR success rate; # number; * significant *p*-value; ^†^ medium effect size (0.3 ≤ *w* < 0.5).

## Data Availability

The data presented in this study are available on request from the corresponding author.

## Supplementary Materials

The following supporting information can be downloaded at: https://www.mdpi.com/article/10.3390/life14040426/s1, Scheme S1: Speech protocol for the audio recording of the mobility and stretching exercises for the active work breaks; Table S1: Task ability requirements; Scheme S2: Self-developed evaluation questionnaire; Table S2: Descriptive results (mean (SD)) of ratings of perceived discomfort for each subgroup; Table S3: Statistical results of the GEE and effect size index *w* for the main and interaction effects of rating of perceived discomfort for the three subgroup analyses; Figure S1: Rating of perceived discomfort at the beginning (T_2_) and end (T_7_) of the simulated laparoscopy for the subgroup analyses: (a) sex with female (light gray) vs. male (dark gray); (b) age with younger (light gray) vs. older (dark gray); (c) job tenure with novices (light gray) vs. experts (dark gray). Figure S2: Boxplots displaying the minimum, first quantile, median, third quantile and maximum of the performance outcomes during the first (WB_1_, light gray), second (WB_2_, middle gray), and third (WB_3_, dark gray) work blocks: (a) number of placed beads in the pick-and-place task; (b) tightened gummies in the pick-and-tighten task; (c) threaded beads in the pick-and-thread task; (d) stick-up points in the pick-and-place task. Figure S3: Perceived workload (NASA TLX) scores for each of the six dimensions and the overall workload. Boxplots displaying the minimum, first quantile, median, third quantile and maximum of the workload scores after the condition without (W; light gray), with passive (P; middle gray), and with active (A; dark gray) work breaks.
