# Supplementary material for: Influence of Intraoperative Active and Passive Breaks in Simulated Minimally Invasive Procedures on Surgeons’ Perceived Discomfort, Performance, and Workload
Source: Life (Basel). 2024 Mar 22;14(4):426. doi: 10.3390/life14040426 (PMC11051257; doi:10.3390/life14040426)
Supplement: Supplementary file 1 [file life-14-00426-s001.zip › Figure_S1_Subgroups.pdf]

Supplementary Material 6

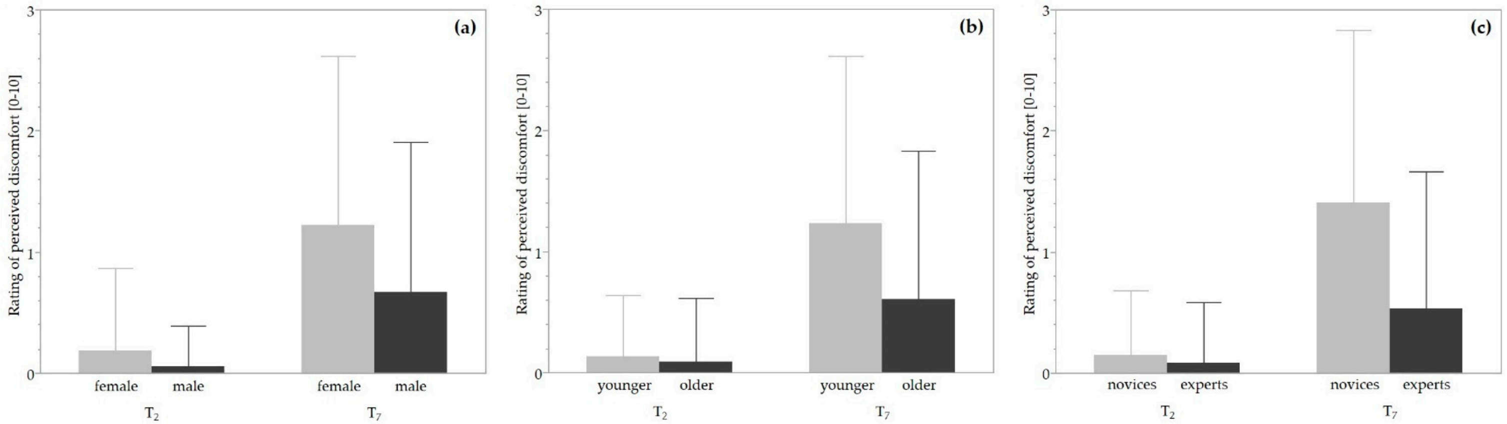

Figure S6. Rating of perceived discomfort at the beginning (T_2) and end (T_7) of the simulation for the subgroup analyses: **(a)** sex with female (light grey) vs. male (dark grey); **(b)** age with younger (light grey) vs. older (dark grey); **(c)** job tenure with novices (light grey) vs. experts (dark grey).
