# Supplementary material for: Influence of Intraoperative Active and Passive Breaks in Simulated Minimally Invasive Procedures on Surgeons’ Perceived Discomfort, Performance, and Workload
Source: Life (Basel). 2024 Mar 22;14(4):426. doi: 10.3390/life14040426 (PMC11051257; doi:10.3390/life14040426)
Supplement: Supplementary file 1 [file life-14-00426-s001.zip › Figure_S2_Tasks_Performance.pdf]

Supplementary Material 7

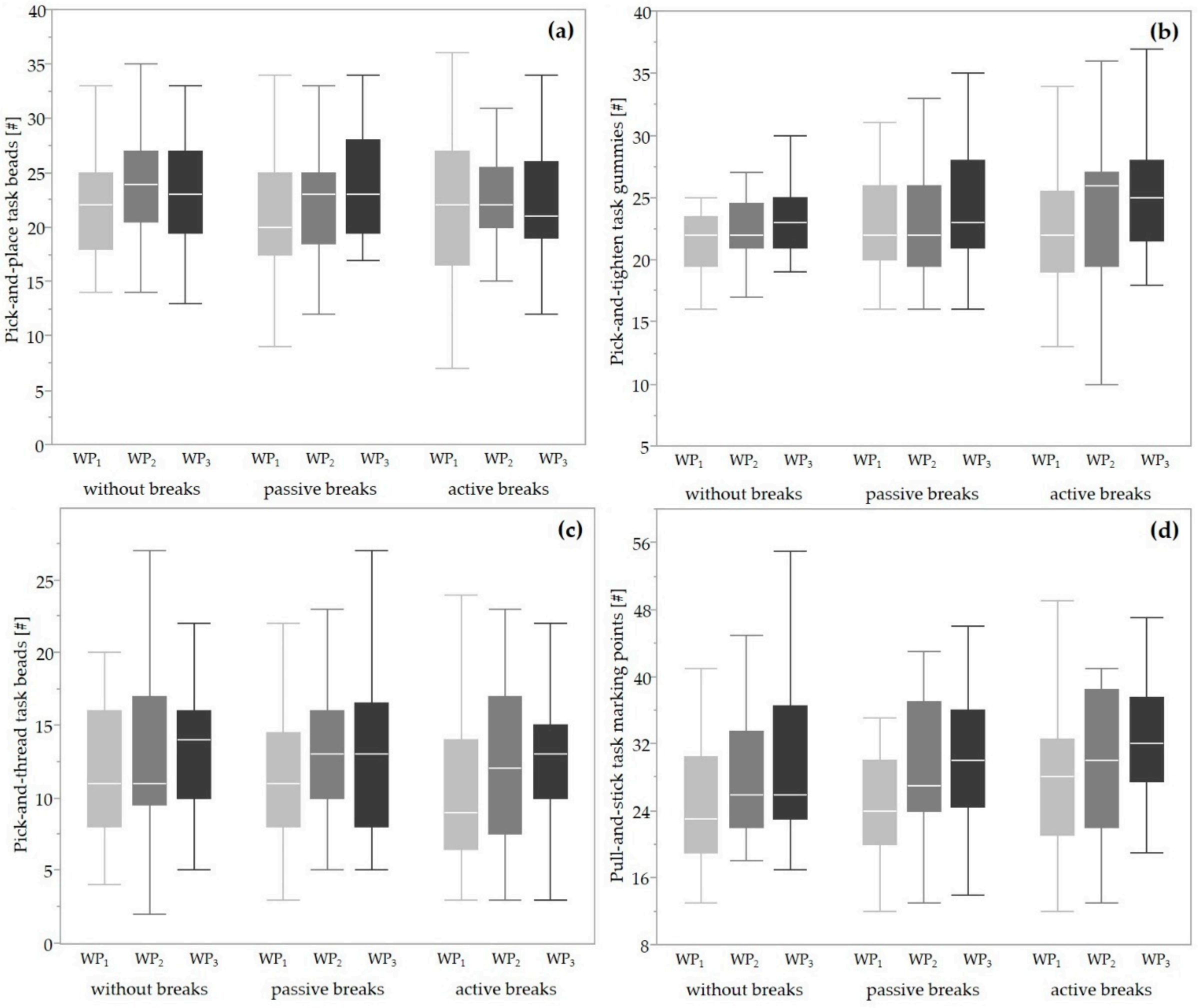

Figure S7. Boxplots displaying minimum, 1st quantile, median, 3rd quantile and maximum of the performance outcomes during the first (WB_1, light grey), second (WB_2, middle grey) and third (WB_3, dark grey) work-blocks: **(a)** number of placed beads in the pick-and-place task; **(b)** tightened gummies in the pick-and-tighten task; **(c)** threaded beads in the pick-and-thread task; **(d)** stick-up points in the pick-and-place task.
