# Supplementary material for: Influence of Intraoperative Active and Passive Breaks in Simulated Minimally Invasive Procedures on Surgeons’ Perceived Discomfort, Performance, and Workload
Source: Life (Basel). 2024 Mar 22;14(4):426. doi: 10.3390/life14040426 (PMC11051257; doi:10.3390/life14040426)
Supplement: Supplementary file 1 [file life-14-00426-s001.zip › Figure_S3_Workload.pdf]

Supplementary Material 8

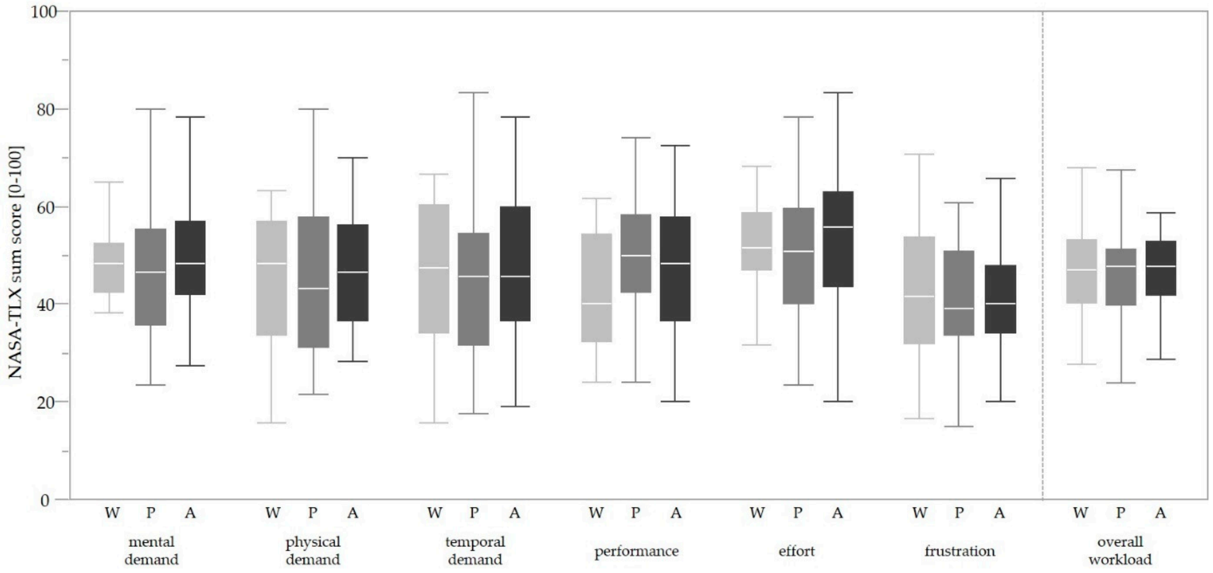

Figure S8. Perceived workload (NASA TLX) scores for each of the six dimensions and the overall workload. Boxplots displaying minimum, 1st quantile, median, 3rd quantile and maximum of the workload scores after the condition without (W; light grey), with passive (P; middle grey) and with active (A; dark grey) work breaks.
