# Supplementary material for: Influence of Intraoperative Active and Passive Breaks in Simulated Minimally Invasive Procedures on Surgeons’ Perceived Discomfort, Performance, and Workload
Source: Life (Basel). 2024 Mar 22;14(4):426. doi: 10.3390/life14040426 (PMC11051257; doi:10.3390/life14040426)
Supplement: Supplementary file 1 [file life-14-00426-s001.zip › Scheme_S1_Transcription_Protocol.pdf]

Supplementary Material 1

Scheme S1. Speech protocol for the audio recording of the mobility and stretching exercises for the active work breaks *(translated from German).*

Starting position that you take during the entire active break: Take a hip-wide standing position and clasp your hands in front of your chest.
Exercise 1: Start walking on the spot without lifting your toes off the floor while slightly rotating your upper body.
Exercise 2: Move your hips alternately from left to right.
Exercise 3: Alternately move your pelvis back and forth. This alternately leads to a light hollow cross or rounded back.
Exercise 4: Bring your hips forward and push your sternum up and pull your shoulders slightly back. Hold the end position for a few seconds.
Exercise 5: Make small backward circular movements in your shoulders.
Exercise 6: Make your neck long by making a light double chin. Hold the end position for a few seconds.
Exercise 7: Turn your head (without force) alternately from left to right.
Exercise 8: Start walking on the spot without lifting your toes off the floor while slightly rotating your upper body.
