# Supplementary material for: Influence of Intraoperative Active and Passive Breaks in Simulated Minimally Invasive Procedures on Surgeons’ Perceived Discomfort, Performance, and Workload
Source: Life (Basel). 2024 Mar 22;14(4):426. doi: 10.3390/life14040426 (PMC11051257; doi:10.3390/life14040426)
Supplement: Supplementary file 1 [file life-14-00426-s001.zip › Scheme_S2_Questionnaire.pdf]

Supplementary Material 3

Scheme S3. Self-developed evaluation questionnaire *(translated from German).*

1 *In which parts of the body did you experience a clear recovery effect during the work breaks? (multiple answers are possible)*

□ fingers

□ wrists

□ shoulder / neck

□ upper back

□ lower back

□ hips

□ knees

□ ankles

□ other: ____________________

2 *How satisfied were you with the content of the work break during the simulated laparoscopy?*

□ very satisfied

□ rather satisfied

□ neither satisfied nor dissatisfied

□ rather dissatisfied

□ very dissatisfied

3 *How do you rate the frequency of the work breaks?*

□ far too many

□ rather too many

□ neither too many nor too few

□ rather too few

□ far too few

4 *How do you rate the duration of the work breaks?*

□ too short

□ a little too short

□ neither too short nor too long

□ a little too long

□ too long

5 *By including work breaks during simulated laparoscopy, my physical performance has:*

□ clearly increased

□ slightly increased

□ neither increased nor decreased

□ slightly reduced

□ clearly reduced

6 *By including relief phases during simulated laparoscopy, my ability to concentrate has:*

□ clearly increased

□ slightly increased

□ neither increased nor decreased

□ slightly reduced

□ clearly reduced

7 *How likely is it that you will carry out short work breaks on your own initiative during a routine operation of at least 1.5 hours?*

absolutely unlikely — absolutely likely (for sure)
