# Supplementary material for: Influence of Intraoperative Active and Passive Breaks in Simulated Minimally Invasive Procedures on Surgeons’ Perceived Discomfort, Performance, and Workload
Source: Life (Basel). 2024 Mar 22;14(4):426. doi: 10.3390/life14040426 (PMC11051257; doi:10.3390/life14040426)
Supplement: Supplementary file 1 [file life-14-00426-s001.zip › Table_S1_Task_Ability_Requirements.pdf]

Supplementary Material 2

Table S2. Task ability requirements.

	**Hot-wire task**	**Peg-transfer**	**Pick-and-place**	**Pick-and-tighten**	**Pick-and-thread**	**Pull-and-stick**
Aiming accuracy	++	+	++	+	++	+
Controlled movement	++	+	++	-	++	+
Controlled force application	-	-	-	++	-	+
Hand-foot coordination	-	-	++	-	+	-
Hand-hand coordination	-	++	+	+	++	+
Fine motor skills	+	-	+	-	++	+
