# Supplementary material for: Influence of Intraoperative Active and Passive Breaks in Simulated Minimally Invasive Procedures on Surgeons’ Perceived Discomfort, Performance, and Workload
Source: Life (Basel). 2024 Mar 22;14(4):426. doi: 10.3390/life14040426 (PMC11051257; doi:10.3390/life14040426)
Supplement: Supplementary file 1 [file life-14-00426-s001.zip › Table_S2_Subgroup_Descriptives.pdf]

Supplementary Material 4

Table S4. Descriptive results (mean (SD)) of rating of perceived discomfort for each subgroup.

Factor		Without Breaks		Passive Breaks		Active Breaks	
		T_2	T_7	T_2	T_7	T_2	T_7
Sex	Women	0.0 (0.0)	1.1 (1.4)	0.2 (0.7)	1.0 (1.3)	0.3 (1.0)	1.6 (1.6)
	Men	0.0 (0.0)	0.8 (1.2)	0.0 (0.0)	0.5 (0.9)	0.2 (0.6)	0.8 (1.6)
Age	Younger (≤ 35 y/o)	0.0 (0.0)	1.4 (1.4)	0.2 (0.6)	1.0 (1.2)	0.2 (0.6)	1.3 (1.7)
	Older (> 35 y/o)	0.0 (0.0)	0.5 (1.0)	0.0 (0.0)	0.5 (1.0)	0.3 (0.9)	0.9 (1.6)
Work experience	Novices (≤ 6 <)	0.0 (0.0)	1.6 (1.3)	0.2 (0.7)	1.2 (1.3)	0.2 (0.7)	1.4 (1.7)
	Experts (> 6 y)	0.0 (0.0)	0.4 (1.0)	0.0 (0.0)	0.3 (0.8)	0.3 (0.9)	0.8 (1.5)
