# Supplementary material for: Influence of Intraoperative Active and Passive Breaks in Simulated Minimally Invasive Procedures on Surgeons’ Perceived Discomfort, Performance, and Workload
Source: Life (Basel). 2024 Mar 22;14(4):426. doi: 10.3390/life14040426 (PMC11051257; doi:10.3390/life14040426)
Supplement: Supplementary file 1 [file life-14-00426-s001.zip › Table_S3_Subgroup_Statistics.pdf]

Supplementary Material 5

Table S5. Statistical results of the GEE and effect size index w for the main and interaction effects of rating of perceived discomfort for the three subgroup analyses.

		**Condition**			**Time**			**Condition x Time**		
		**χ^2 (df)**	*p*	*w*	**χ^2 (df)**	*p*	*w*	**χ^2 (df)**	*p*	*w*
**Sex**	**Women**	2.257 (2)	0.324	0.204	8.820 (1)	**0.003***	0.404†	7.200 (2)	**0.027***	0.365†
	**Men**	1.969 (2)	0.428	0.153	4.495 (1)	**0.034***	0.250	1.500 (2)	0.472	0.144
**Age**	**Younger (≤ 35 y/o)**	0.368 (2)	0.832	0.078	10.879 (1)	**0.000***	0.426†	4.286 (1)	0.117	0.267
	**Older (> 35 y/o)**	2.444 (2)	0.295	0.192	3.257 (1)	0.071	0.222	2.444 (2)	0.295	0.192
**Work experience**	**Novices (≤ 6 <)**	0.125 (2)	0.940	0.044	9.561 (1)	**0.002***	0.381†	2.933 (2)	0.231	0.211
	**Experts (> 6 y)**	2.319 (2)	0.314	0.197	3.536 (1)	0.060	0.243	4.286 (2)	0.117	0.267

* significant *p*-value; † medium effect size ($0.3 \leq w < 0.5$).
