# Supplementary figures and images for: Influence of Intraoperative Active and Passive Breaks in Simulated Minimally Invasive Procedures on Surgeons’ Perceived Discomfort, Performance, and Workload
Source: Life (Basel). 2024 Mar 22;14(4):426. doi: 10.3390/life14040426 (PMC11051257; doi:10.3390/life14040426)

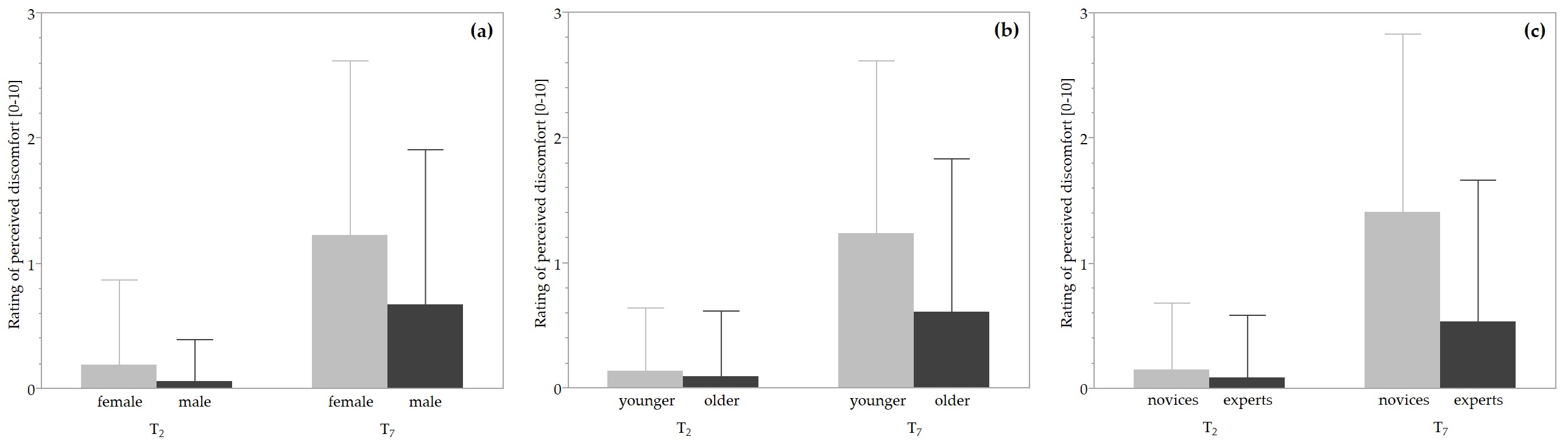

Supplement: Supplementary file 1 [file life-14-00426-s001.zip › Figure_S1_Subgroups.jpg]

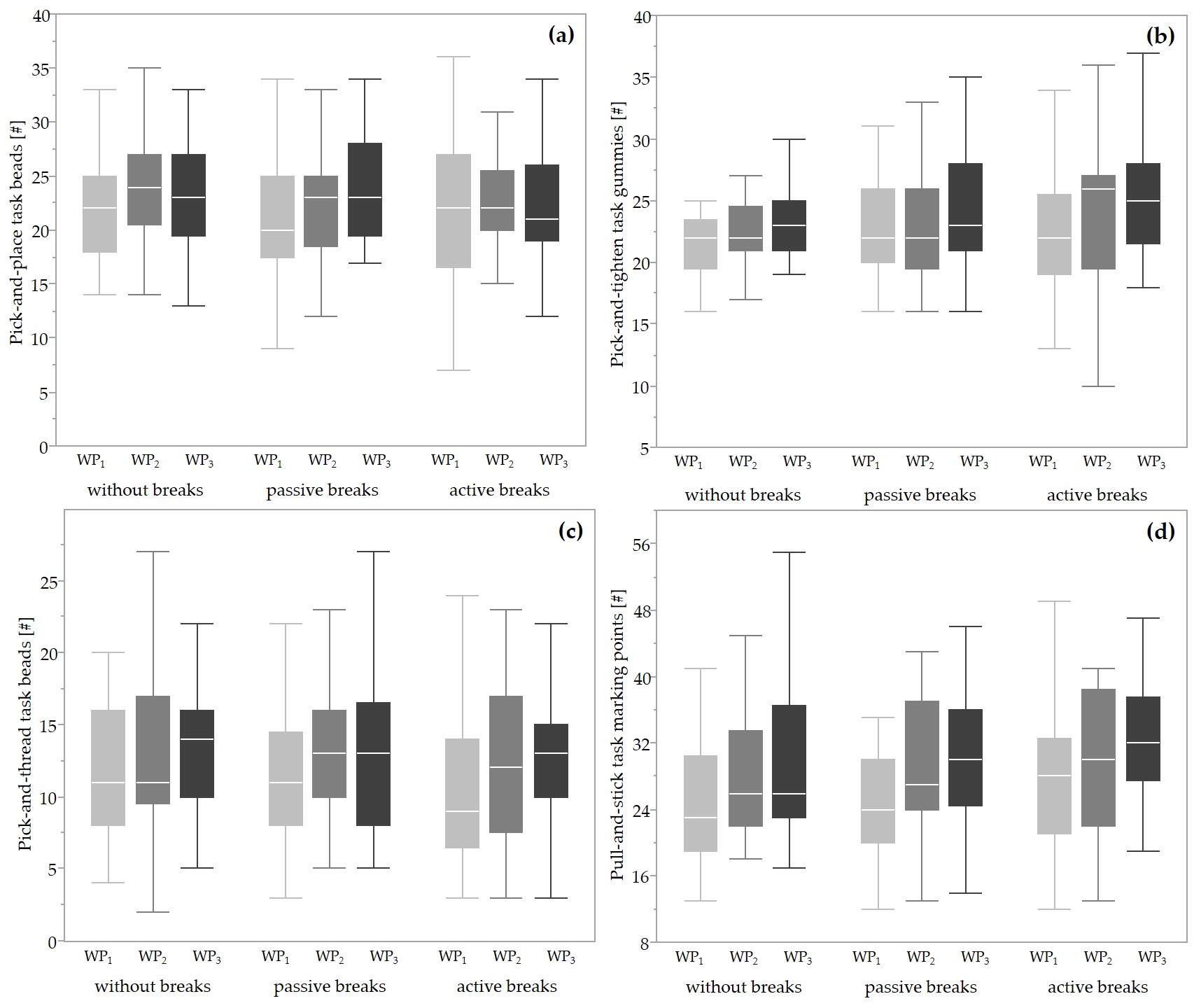

Supplement: Supplementary file 1 [file life-14-00426-s001.zip › Figure_S2_Performance_4Tasks.jpg]

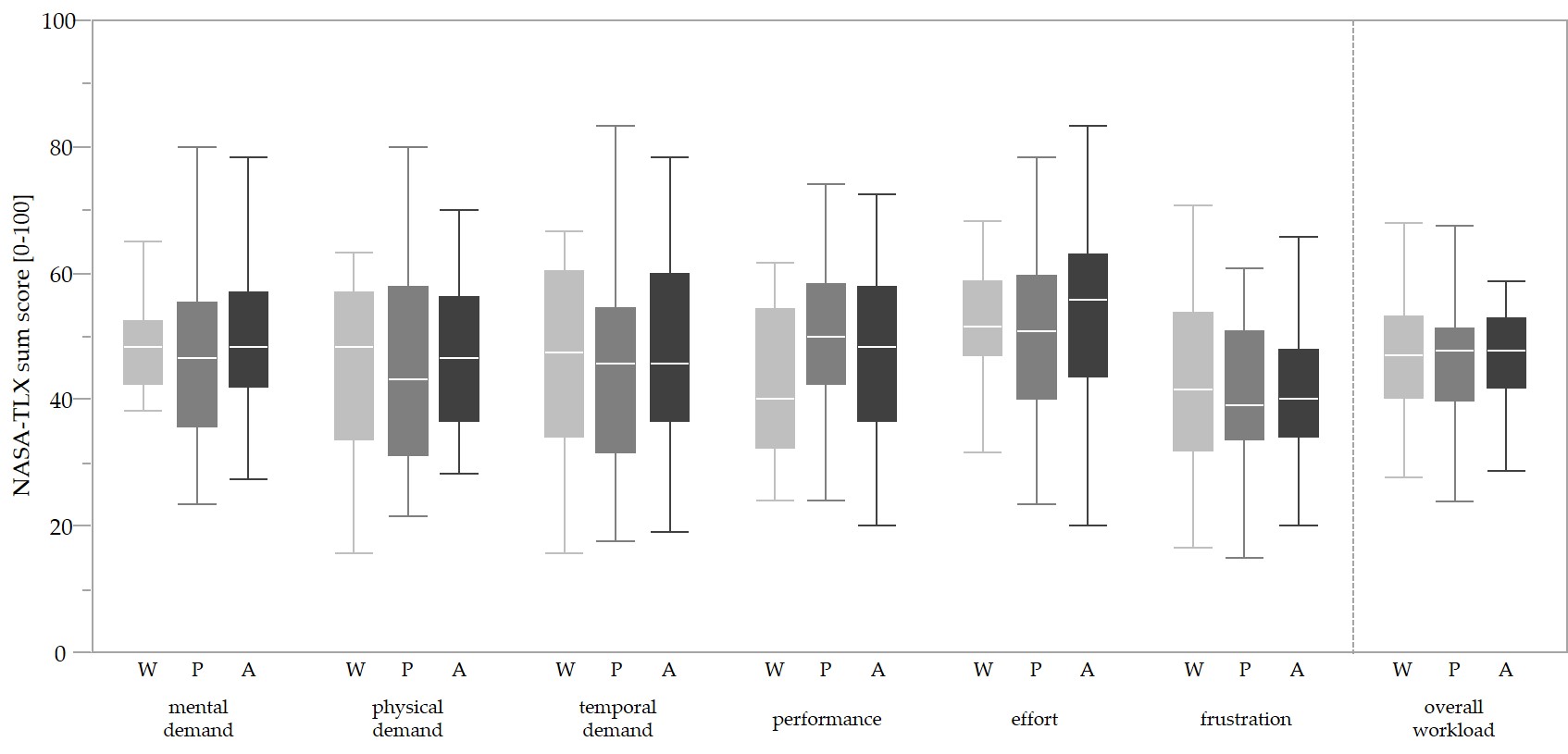

Supplement: Supplementary file 1 [file life-14-00426-s001.zip › Figure_S3_Workload.jpg]
